# Towards an effective control programme of soil-transmitted helminth infections among Orang Asli in rural Malaysia. Part 2: Knowledge, attitude, and practices

**DOI:** 10.1186/1756-3305-6-28

**Published:** 2013-01-28

**Authors:** Nabil A Nasr, Hesham M Al-Mekhlafi, Abdulhamid Ahmed, Muhammad Aidil Roslan, Awang Bulgiba

**Affiliations:** 1 Department of Parasitology, Faculty of Medicine, University of Malaya, Kuala Lumpur, 50603, Malaysia; 2 Department of Biology, Faculty of Natural and Applied Sciences, Umaru Musa Yar’adua University, Katsina, Katsina State, Nigeria; 3 Julius Centre University of Malaya, Department of Social & Preventive Medicine, Faculty of Medicine, University of Malaya, Kuala Lumpur, 50603, Malaysia; 4 Department of Parasitology, Faculty of Medicine and Health Sciences, Sana’a University, Sana’a, Yemen

**Keywords:** Knowledge, Attitude, Practice, Soil-transmitted helminths, Malaysia

## Abstract

**Background:**

In the first part of this study, we investigated the prevalence and associated key factors of soil-transmitted helminth (STH) infections among Orang Asli children in rural Malaysia; an alarming high prevalence and five key factors significantly associated with infections were reported. Part 2 of this study aims to evaluate the knowledge, attitude and practices (KAP) on STH infections among Orang Asli in Peninsular Malaysia.

**Methods:**

A cross-sectional study was carried out among 215 households from 13 villages in Lipis district, Pahang, Malaysia. Demographic and socioeconomic information of the participants and their KAP on STH were collected by using a pre-tested questionnaire.

**Results:**

Overall, 61.4% of the participants had prior knowledge about intestinal helminths with a lack of knowledge on the transmission (28.8%), signs and symptoms (29.3%) as well as the prevention (16.3%). Half of the respondents considered STH as harmful, while their practices to prevent infections were still inadequate. Significant associations between the KAP and age, gender, educational and employment status, family size, and household monthly income were reported. Moreover, significantly lower prevalence of STH infections was reported among children of respondents who wear shoes/slippers when outside the house (72.8%; 95% CI= 62.6, 80.5 vs 87.0%; 95% CI= 81.4, 91.1), wash their hands before eating (32.4%; 95% CI= 24.3, 42.2 vs 51.4%; 95% CI= 44.7, 60.1), and wash their hands after defecation (47.8%; 95% CI= 35.7, 57.1 vs 69.2%; 95% CI= 63.7, 78.7) as compared to their counterparts. Multiple logistic regression analysis indicated that the educational level of the respondents was the most important factor significantly associated with the KAP on STH among this population.

**Conclusion:**

This study reveals inadequate knowledge, attitude and practices on STH infections among Orang Asli in rural Malaysia. Hence, there is a great need for a proper health education programme and community mobilisation to enhance prevention and instil better knowledge on STH transmission and prevention. This is crucial for an effective and sustainable STH control programme to save the lives and future of the most vulnerable children in rural Malaysia.

## Background

Soil-transmitted helminth (STH) infections, among the most common neglected tropical diseases (NTDs), are major public health problems in developing countries particularly among children in rural areas with more than two-thirds of the cases occur in Asia [1,2]. These infections, mainly by the triad of *Ascaris lumbricoides*, hookworm (*Ancylostoma duodenale* and *Necator americanus*) and *Trichuris trichiura* are among the most important predictors of malnutrition, micronutrient deficiencies, poor cognitive function, school absenteeism and a dismal academic performance among children [3-7]. Moreover, the devastating impact of STH infections may affect the economic productivity and trap endemic communities in a cycle of poverty, underdevelopment and disease [8-10].

In the eternal battle of humans against worms, a few stories of success in eliminating and reducing the transmission, prevalence and intensity of STH infections have been documented in South Korea, China and Japan [11-13], with mass de-worming, proper sanitation and hygiene education being the main components of control programmes. Despite the potential threat of anthelmintics drug resistance developing in the STH [14,15], mass drug administration has been used for generations as the main pillar and the most cost-effective intervention to control STH. In addition, adequate sanitation plays an important role in protecting the uninfected individuals and reducing the environmental sources of infections.

Moreover, health education that is effective, targeted and simple is often recommended as a first option to create the enabling environment for other strategies to thrive, especially in underprivileged communities [16,17]. In the same vein, the participation of the community represents one of the cardinal tools of disease control programmes as improvements in the awareness and understanding can greatly increase the realisation and sustainability of long-term STH control strategies. Furthermore, the knowledge and practices of targeted people were found to be instrumental in designing and implementing effective community-based de-worming programmes [18].

In Malaysia, the trend of STH infections in rural areas has remained largely unchanged since the 1920s, with alarming high prevalence and prominent morbidity reported among Orang Asli children in rural Malaysia [19-21]. However, information on the knowledge, attitude and practices (KAP) of these infections is lacking and providing such information is important for implementing integrated and effective control measures, and also for planning, implementation and evaluation of advocacy, communication and social mobilisation work. Hence, this study aimed at evaluating the KAP of Orang Asli people about STH infections. It is expected that this information will assist public health officials to design an effective and integrated STH control programme.

## Methods

### Study area and study population

A cross-sectional study was carried out in the Lipis district of Pahang state, located at the center of Peninsular Malaysia, about 200 km northeast of Kuala Lumpur with a total area of 5,198 km^2^ and a total population of 87,200 people (2010 census). Data collection was carried out over a period of six months, from April to September 2011. This study was conducted in 13 Orang Asli villages (Figure 1 of part 1) [22]. The probability proportional to size sampling method was used to select 215 households from 710 households in these villages. Moreover, 484 children (51.4% males and 48.6% females) aged ≤ 15 years and living in the selected households had agreed voluntarily to participate in the parasitological survey.

### Questionnaire survey

This survey utilized a validated structured questionnaire to collect information on the KAP of the participants towards intestinal helminth infections as well as their demographic, socioeconomic and environmental information, and health status (details on the design and validation of the questionnaire are provided in Part 1) [22]. Questions on knowledge were open-ended questions, without multiple-choice answers to avoid guessing which may give a false impression of the knowledge of the participant. On the other hand, the questions on the practices were provided with multiple-choice answers to assess the frequency of doing these activities or actions. Similarly, questions on the attitude were also with multiple-choice answers to evaluate the prevailing attitudes, beliefs and misconceptions that the participants may have about intestinal helminth infections.

At their home setting, the heads of the households were interviewed by two health assistants from JAKOA and from the Department of Parasitology, University of Malaya. Both assistants were trained on the purpose of the study and on how to administer the questionnaire. During the interviews, observations were made by another researcher on the personal hygiene of the children and household cleanliness including the availability of functioning toilets, piped water, cutting nails, wearing shoes when outside the house, hands and clothes cleanliness.

### Parasitology

Fresh faecal samples were collected in clearly labeled containers with wide mouth and screw-caps. The samples were examined for the presence of intestinal helminths including STH eggs or larvae by three different techniques, formalin-ether sedimentation, Kato Katz, and Harada Mori culture techniques. Description of parasitological examination is provided in part 1 of this study [22].

### Data analysis

Double data entry was done by two different researchers into Microsoft Office Excel 2007 spreadsheets, and the two data sets were then cross-checked for accuracy by a third researcher. Data analysis was performed by using SPSS version 13. The demographic and socioeconomic characteristics of the respondents were treated as categorical variables and presented as frequencies and percentages. We also recoded all the KAP items into dichotomous variables to ease interpretation. For example, if an item has four response options (always, sometimes, rarely, and never), then it was dichotomized into always/sometimes versus rarely/never).

For data analysis, the dependent variables were the components or variables of the KAP, while the independent variables were the demographic and socioeconomic factors, and the STH infections status. Pearson’s Chi Square test and Fisher’s Exact test were used to show whether the difference between the groups of comparison is significant or otherwise, and also to test the associations between the variables; odd ratios (OR) and 95% confidence intervals (CI) were computed. In order to control the variation in number of children in households, weight cases, derived by the sampling fraction 1/ number of participated children from each family, was used to analyse the data for the association between the KAP and infection status among children. A *P* value of ≤ 0.05 was considered statistically significant. Multiple logistic regression analysis was performed to identify the factors significantly associated with KAP variables; a *P* value of 0.25 was used as a screening criterion for the selection of variables to the logistic regression model [23].

### Ethical consideration

The protocol of this study was approved by the Medical Ethics Committee of the University of Malaya Medical Centre, Malaysia (Reference Number: 788.74). During fieldwork, the objectives and procedures of the study were explained to the heads of the sampled households. They were informed that their participation was totally voluntary and that they could decide to withdraw from the study at any time without giving any reason whatsoever. Written and signed or thumb-printed informed consents were obtained from all adult participants before starting the survey. Similarly, written and signed or thumb-printed informed consents were taken from parents or guardians, on behalf of their children. All the infected children were treated with a single dose of albendazole 400 mg.

## Results

### General characteristics of the households

A total of 215 households from 13 villages in Lipis, Pahang participated in this study and heads of the households were interviewed face-to-face to investigate their KAP towards intestinal helminth parasites. Out of the 215 respondents, 125 (58.1%) were females and 90 (41.9%) were males with a mean age of 33.1±9.2 years. About two thirds of the males had at least 6 years of formal education with 16.3% of them having gone through secondary education. On the other hand, about half of the females had primary education while 17.7% had secondary education. Moreover, about one third of the households had low monthly income (<RM500). Most of the houses were made of wood or bamboo and about half of the houses had piped water supply (gravity-fed) and 89.8% had electricity. Most of the residents worked as labourers in the palm oil or rubber plantations while about 5% were farmers.

### Prevalence and distribution of STH infections

A total of 484 children aged ≤ 15 years (51.4% males and 48.6% females) and living in the sampled households had delivered faecal samples for examination. Overall, 378 (78%) children were found to be infected with at least one species of STH. The predominant species was *T. trichiura* with a prevalence rate of 71.7%, followed by *A. lumbricoides* (37.4%) and then hookworms (17.6%). Data on the prevalence and key factors significantly associated with STH are provided in part 1 of this study [22].

### Knowledge about intestinal helminths, their signs and symptoms, transmission and prevention

A total of 215 Orang Asli householders were interviewed face-to-face to fill in the questionnaire on their KAP towards intestinal helminths infections. The general results of the knowledge of Orang Asli participants about intestinal helminths transmission, signs and symptoms and prevention are shown in Table 1. It was found that 132 (61.4%) participants had heard about the intestinal worms. Out of these 132 participants, 27 (20.3%) indicated that the main source of their information about worms was the clinic, while 83 (63.1%) could not remember the source of information. When the participants were asked about the types of intestinal helminths they know, only 10 (7.6%) mentioned pinworm and 7 (5.3%) mentioned roundworms. As a misconception, 2 (1.5%) of them mentioned earthworm as a type of intestinal worm. The results further showed that there was a lack of knowledge of the signs and symptoms of intestinal helminth infections among Orang Asli people where only 29.3% (63/215) of the respondents were able to mention at least one symptom of intestinal helminth infections. Of those, 36.5% (23/63) and 39.7% (25/63) mentioned one and two symptoms, respectively. The main signs and symptoms were abdominal pain and abdominal distension followed by diarrhoea, loss of appetite, and vomiting.

**Table 1:** Knowledge about intestinal helminths, symptoms, transmission and prevention among Orang Asli who had prior knowledge on intestinal helminths (n = 132)

**Variable**	**N**	**(%)**
**Source of information**		
Health clinic/hospitals	27	20.5
Health workers/sanitary inspectors	14	10.6
Mass media	3	2.3
Other people	3	2.3
School	2	1.5
Do not remember	83	62.9
**Signs and symptoms**		
Abdominal pain	21	33.3
Abdominal distention	21	33.3
Diarrhoea	19	30.2
Vomiting	15	23.8
Loss of appetite	16	25.4
Pale face	12	19.0
Body weakness	10	15.9
**Transmission**		
Eating contaminated food	17	27.4
Dirty hands	15	24.2
Walking barefooted	15	24.2
Drinking untreated water	7	11.3
Playing with soil	12	19.4
Not cutting nails regularly	6	9.7
Eating soil (geophagy)	5	8.1
**Prevention**		
Taking de-worming drugs	18	13.6
Washing of hands before eating	16	12.1
Wearing of shoes when outside the house	13	9.8
Boiling of untreated drinking water	4	3.0

Besides that, 28.8% (62/215) of the respondents have knowledge of the ways of transmission of intestinal helminths. Of them, 17 (27.4%) respondents mentioned eating contaminated food while 15 (24.2%) respondents mentioned dirty hands and walking barefooted each. On the other hand, only 16.3% (35/215) of the respondents had knowledge about the prevention of helminth infections. Of them, 16 and 13 participants mentioned hand washing before eating and wearing shoes/slippers when outside the house, respectively.

### Attitude and practices of Orang Asli people to intestinal helminths

Results about the attitude and practices of Orang Asli people towards intestinal helminths are illustrated in Table 2. Out of the 132 participants with prior knowledge on intestinal worms, 71 (53.6%) considered the intestinal helminths as harmful to people’s health. Interestingly, one participant held the view that intestinal helminths are good for people’s health.

**Table 2:** Attitude and perceived practices towards intestinal helminths among Orang Asli in Lipis, Pahang, Malaysia (n = 215)

**Variables**	**N**	**%**
**Effects of intestinal helminths**		
Harmful to peoples’ health	71	33.0
Beneficial to peoples’ health	1	0.5
Do not know	143	66.5
**Faeces as source of infections**		
Yes	65	30.3
No	9	4.2
Do not know	141	65.5
**Practices**		
Hand washing before eating	97	45.2
Hand washing after defecation	134	62.3
Wearing shoes when outside the house	169	78.8
Cutting fingernails regularly	118	54.8
Washing vegetables before eating	148	68.9
Washing of fruits before eating	88	41.0
Boiling untreated drinking water	59	27.4
Seeking treatment for diarrhoea and abdominal pain from clinic	214	99.5

Regarding the practices, the results revealed that 118 (54.8%) respondents do not wash their hands before having their meals, and normally eat using their hands. Similarly, 81 (37.7%) of the respondents do not wash their hands after defecation while about half of them 100 (46.5%) walk barefooted. Moreover, about one third (31.1%) of the respondents do not wash fruits/vegetables before consumption and 97 (45.2%) of them do not cut their nails regularly. In terms of treatment-seeking behaviour, almost all (99.1%) of the participants mentioned that they go to the nearest clinic for treatment in case of diarrhoea and abdominal pain, while just one participant mentioned that he goes to a traditional healer.

### Associations of knowledge of Orang Asli participants on intestinal helminth infections with some demographic and socioeconomic factors

Associations of knowledge about intestinal helminth infections with the age and gender of the respondents are shown in Table 3. Knowledge of abdominal pain and abdominal distension as symptoms of STH infections was significantly higher in males than females (*P* = 0.050). Similarly, the males had significantly higher level of knowledge about the preventive measures (*P* = 0.045), and about taking de-worming drugs (*P* = 0.026) than the females. Moreover, the respondents aged < 32 years had significantly less knowledge about diarrhoea than those aged ≥ 32 years (4.8% vs 12.7%; *P* = 0.040).

**Table 3:** Associations of knowledge of Orang Asli participants about intestinal helminths with age and gender

**Variable**	**Age (years)**				**Gender**			
	**< 32 years**	**>= 32 years**	**OR**	**95% CI**	**Male**	**Female**	**OR**	**95% CI**
–Heard about worms	59(56.2)	73(66.4)	0.7	0.4, 1.1	56(62.2)	76 (60.8)	1.1	0.6, 1.9
**Signs and symptoms:**								
–At least one symptom	28(26.7)	35(31.8)	0.8	0.4, 1.4	30(33.3)	33(26.4)	1.4	0.8, 2.5
–Abdominal pain	7(6.7)	14(12.7)	0.5	0.2, 1.3	13(14.4)	8(6.4)	2.4	1.0, 6.2*
–Abdominal distension	10(9.5)	11(10.0)	0.9	0.4, 2.3	13(14.4)	8(6.4)	2.4	1.0, 6.2*
–Vomiting	6(5.7)	9(8.2)	0.7	0.2, 1.9	6(6.7)	9(7.2)	0.9	0.3, 2.7
–Diarrhoea	5(4.8)	14(12.7)	0.3	0.1, 0.9*	11(12.2)	8(6.4)	2.0	0.8, 5.3
–Lack of appetite	6(5.7)	10 (9.1)	0.6	0.2, 1.7	7(7.8)	9(7.2)	1.1	0.4, 3.0
–Pale face	5(4.8)	7(6.4)	0.7	0.2, 2.4	4(4.4)	8(6.4)	0.7	0.2, 2.3
**Transmission:**								
–At least one way of transmission	29(27.6)	33(30.0)	0.9	0.5, 1.6	29(32.2)	32(25.8)	1.4	0.8, 2.5
–Playing with soil	6(5.7)	6(5.5)	1.1	0.3, 3.4	7(7.8)	5(4.0)	0.5	0.2, 1.6
–Stagnant water	6(5.7)	8(7.3)	0.8	0.3, 2.3	9(10.0)	5(4.0)	2.6	0.8, 7.2
–Dirty hands	4(3.8)	11(10.0)	0.4	0.1, 1.2	4 (4.4)	11(8.8)	0.4	0.1, 1.6
–Walking barefooted	5(4.8)	10(9.1)	0.5	0.2, 1.5	6(6.7)	9(7.2)	0.9	0.3, 2.7
–Eating contaminated food	11(10.5)	6(5.5)	2.0	0.7, 5.7	10(11.1)	7(5.6)	2.1	0.8, 5.7
**Prevention:**								
–At least one preventive measure	13(12.4)	22 (20)	0.6	0.3, 1.2	20(22.2)	15(12.0)	2.1	1.0, 4.4*
–Washing hands before eating	6(5.7)	10(9.1)	0.6	0.2, 1.7	9(10.0)	7(5.6)	1.8	0.7, 5.2
–Wearing shoes when outside the house	4(3.8)	9(8.2)	0.4	0.1, 1.5	7(7.8)	6(4.8)	1.7	0.5, 5.1
–Taking de-worming drugs	8(7.6)	10(9.1)	0.8	0.3, 2.2	12(13.3)	6(4.8)	3.1	1.1, 6.4*

All values are number (%). OR, Odds ratio. CI, Confidence interval. * Significant association (*P*<0.05).

Table 4 shows that the educated respondents showed significantly higher level of knowledge about the worms and their signs and symptoms (39.2%; 95% CI= 31.3, 47.8) than the non-educated respondents (14.1%; 95% CI= 8.3, 23.1). The percentage of the educated respondents who had heard about the intestinal worms (66.9%; 95% CI= 61.2, 74.4) was significantly higher when compared to those who had not heard about the worms (52.9%; 95% CI= 42.4, 59.5). Similarly, respondents who had at least 6 years of formal education had significantly higher level of knowledge of the ways of transmission (*P* = 0.031), the prevention of intestinal helminths infections (*P* = 0.003), and taking de-worming drugs as a preventive measure (*P* = 0.038) compared to the non educated respondents. On the other hand, the results of the current study demonstrated significant associations between the respondents’ employment status and knowledge of abdominal distension (*P* = 0.024) and diarrhoea (*P* = 0.046) as signs and symptoms of intestinal worm infections. Similar associations were found between employment status and knowledge of stagnant water (*P* < 0.001) and eating contaminated food (*P* = 0.003) as ways of transmission of intestinal worm infections.

**Table 4:** Associations of knowledge of Orang Asli participants about intestinal helminths with educational level and employment status

**Variable**	**Educational level**				**Employment status**			
	**Educated**	**Non educated**	**OR**	**95% CI**	**Working**	**Not working**	**OR**	**95% CI**
–Heard about worms	87(66.9)	45(52.9)	1.8	1.0, 3.2*†	30(71.4)	102(59)	1.7	0.8, 3.6
**Signs and symptoms:**								
–At least one symptom	51(39.2)	12(14.1)	3.9	1.9, 7.9*†	17(40.5)	46(26.6)	1.9	0.9, 3.8
–Abdominal pain	18(13.8)	3(3.5)	3.4	1.2, 5.4*	7(16.7)	14(8.1)	1.3	0.9, 4.6
–Abdominal distension	16(12.3)	5(5.9)	2.2	0.8, 6.4	8 (19.0)	13(7.5)	2.8	1.1, 7.2*
–Vomiting	12(9.2)	3(3.5)	2.8	0.8, 6.2	4 (9.5)	11(6.4)	1.6	0.5, 3.5
–Diarrhoea	17(13.1)	2(2.4)	4.5	1.4, 7.7*†	7(16.7)	12(6.7)	2.6	1.0, 7.3*
–Lack of appetite	14(10.8)	2(2.4)	4.0	1.1, 6.2*†	5(11.9)	11(6.4)	1.9	0.7, 6.1
–Pale face	9(6.9)	3(3.5)	2.0	0.5, 7.7	3(7.1)	9(5.2)	1.4	0.4, 4.5
**Transmission:**								
–At least one way of transmission	44(33.8)	17(20.2)	2.0	1.1, 3.8*	21(50.0)	41(23.7)	3.2	1.6, 6.5*†
–Playing with soil	9(6.9)	3(3.5)	2.0	0.5, 7.7	4(9.5)	8(4.6)	2.2	0.6, 5.8
–Stagnant water	11(8.5)	3(3.5)	2.5	0.7, 9.3	9(21.4)	5(2.9)	6.1	2.9, 11.7*†
–Dirty hands	11(8.5)	4(4.7)	1.9	0.6, 6.1	2(4.8)	13(7.5)	0.6	0.1, 2.8
–Walking barefooted	12(9.2)	3(3.5)	2.8	0.8, 7.8	4(9.5)	11(6.4)	1.5	0.5, 5.1
–Eating contaminated food	11(8.5)	6(7.1)	1.2	0.4, 3.4	8(19.0)	9(5.2)	4.2	1.5, 9.8*†
**Prevention:**								
–At least one preventive measure	29(22.3)	6(7.1)	3.8	1.4, 9.5*†	9(21.4)	26(15.0)	1.5	0.7, 3.5
–Washing hands before eating	13(10)	3(3.5)	3.0	0.8, 9.1	5(11.9)	11(6.4)	1.9	0.7, 6.1
–Wearing shoes when outside the house	11(8.5)	2(2.4)	3.8	0.8. 8.6	4 (9.5)	9(5.2)	1.9	0.6, 5.6
–Taking de-worming drugs	15(11.5)	3(3.5)	3.5	1.1, 9.2*†	5(11.9)	13(7.5)	1.7	0.6, 4.9

All values are number (%). OR, Odds ratio. CI, Confidence interval. * Significant association (*P*<0.05). † Variables were confirmed by multiple logistic regression analysis as significant associated factors.

Results in Table 5 further demonstrated that household monthly income was significantly associated with knowledge of the transmission of intestinal helminths (*P* = 0.033), with those having household monthly income of ≥ RM500 having higher level of knowledge. A similar association was reported between household monthly income and knowledge of the prevention of STH infections (*P* = 0.010). Moreover, respondents who live in large families (≥ 7 members) had significantly better knowledge of the signs and symptoms of infections, particularly abdominal pain (*P* = 0.036), vomiting (*P* = 0.011) and diarrhoea (*P* = 0.040), as compared to those who live in small families. Similarly, those who live in large families had significantly higher level of knowledge of the prevention of infections compared to those who live in small families (23.1% vs 12.4%; *P* = 0.042).

**Table 5:** Associations of knowledge of Orang Asli participants about intestinal helminths with family size and household monthly income

**Variable**	**Family size**				**Household monthly income**			
	**>= 7 members**	**< 7 members**	**OR**	**95% CI**	**>= RM500**	**< RM500**	**OR**	**95% CI**
–Heard about worms	67(63.2)	65(59.6)	1.1	0.7, 2.0	43(66.2)	89(59.3)	1.3	0.7, 2.5
**Signs and symptoms:**								
–At least one symptom	36(34.0)	27(24.8)	1.6	0.9, 2.8	24(36.9)	39(26.0)	1.7	0.9, 3.1
–Abdominal pain	12(15.4)	9(6.6)	2.5	1.0, 6.4*	12(18.5)	9(6.0)	3.5	1.4, 8.7*†
–Abdominal distension	9(11.3)	12(8.3)	1.4	0.5, 3.4	7(10.8)	14(9.3)	1.2	0.5, 3.1
–Vomiting	10(12.8)	5(3.6)	3.8	1.3, 9.8*	5 (7.7)	10(6.7)	1.2	0.4, 3.5
–Diarrhoea	11(14.1)	8(5.8)	2.6	1.1, 6.8*	7 (10.8)	12 (8.0)	1.4	0.5, 3.7
–Lack of appetite	8(7.3)	8(7.5)	1.0	0.4, 2.8	5 (7.7)	11(7.3)	1.1	0.4, 3.1
–Pale face	7(9.0)	5(3.6)	2.1	0.7, 7.3	2 (3.1)	10(6.7)	0.4	0.1, 2.1
**Transmission:**								
–At least one way of transmission	26(24.5)	35(32.1)	0.7	0.3, 1.2	25(38.5)	36(24.2)	2.0	1.1, 3.7*
–Playing with soil	5(6.4)	7(5.1)	1.3	0.4, 4.1	4(6.2)	8(5.3)	0.9	0.2, 2.9
–Stagnant water	4(5.1)	10(7.3)	0.7	0.2, 2.2	5(7.7)	9(6.0)	1.3	0.4, 3.7
–Dirty hands	7(6.6)	8(7.3)	0.9	0.3, 2.6	7(10.8)	8(5.3)	2.1	0.7, 5.7
–Walking barefooted	7(6.6)	8(7.3)	0.9	0.3, 2.6	6(9.2)	9(6.0)	1.6	0.5, 4.6
–Eating contaminated food	6(5.7)	11(10.1)	0.5	0.2, 1.5	10(15.4)	7(4.7)	3.7	1.3, 9.7*†
**Prevention:**								
–At least -one preventive measure	18(23.1)	17(12.4)	2.1	1.0, 4.4*	17(26.2)	18(12.0)	2.6	1.2, 5.4*
–Washing hands before eating	6(5.7)	10(9.2)	0.6	0.2, 1.7	9(13.8)	7(4.7)	3.2	1.2, 9.2*
–Wearing shoes when outside the house	7(6.6)	6(5.5)	1.2	0.4, 3.7	6(9.2)	7(4.7)	2.1	0.7, 6.4
–Taking de-worming drugs	12(11.3)	6(5.5)	2.2	0.8, 6.1	8(12.3)	10(6.7)	1.9	0.7, 5.2

All values are number (%). RM, Malaysian Ringgit; (US$1 = RM3.15). OR, Odds ratio. CI, Confidence interval. * Significant association (*P*<0.05). † Variables were confirmed by multiple logistic regression analysis as significant associated factors.

### Associations of attitude and practices of Orang Asli participants towards intestinal helminth infections with some demographic and socioeconomic factors

Table 6 shows that the association of the attitude towards intestinal helminths with the age and gender of the respondents was not significant (*P* > 0.05). On the other hand, practising hand washing before eating was found to be significantly higher in males than females (53.3% vs 39.2%; *P* = 0.040). A similar association was found between the gender and hand washing after defecation (*P* = 0.024).

**Table 6:** Associations of attitude and practices of Orang Asli participants towards intestinal helminth infections with age and gender

**Variable**	**Age**				**Gender**			
	**< 32 years**	**≥ 32 years**	**OR**	**95% CI**	**Male**	**Female**	**OR**	**95% CI**
**Attitude:**								
–Harmful to people’s health	42(38.2)	29(27.9)	1.5	0.9, 2.8	29(32.2)	42(33.9)	0.9	0.5, 1.6
**Practices:**								
–Washing hands before eating	48(43.6)	49(46.7)	0.9	0.5, 1.5	48(53.3)	49(39.2)	1.8	1.0, 3.1*†
–Washing hands after defecation	63(57.3)	71(67.6)	0.6	0.4, 1.1	64(71.1)	70(56.0)	1.9	1.1, 3.4*†
–Wearing shoes when outside the house	89(80.9)	80(76.2)	1.3	0.7, 2.5	97(77.6)	72(80.9)	1.2	0.6, 2.4
–Washing fruits before consumption	49(44.5)	39(37.1)	1.4	0.8, 2.3	38(42.2)	50(40.0)	1.1	0.6, 1.9
–Washing vegetables before consumption	72(65.5)	76(72.4)	0.7	0.4, 1.3	65(72.2)	83(66.4)	1.3	0.7, 2.4
–Boiling drinking water	33(30.0)	26(24.8)	1.3	0.7, 2.4	30(33.3)	29(23.2)	1.7	0.9, 3.0

All values are number (%). OR, Odds ratio. CI, Confidence interval. * Significant association (*P*<0.05). † Variables were confirmed by multiple logistic regression analysis as significant associated factors.

Table 7 shows a significant association between the educational level and the practice of hand washing before eating (*P* = 0.039), with the educated respondents washing their hands more than the non-educated respondents (50.8% vs 36.5%). A similar association was found between the educational level of the respondents and the practice of washing fruits before consumption (*P* = 0.027). On the other hand, a significant association between the employment status of the respondents and the practice of washing fruits before consumption was reported (*P* = 0.042). Overall, the associations of attitude and practices with family size and household monthly income were not significant (*P* > 0.05).

**Table 7:** Associations of attitude and practices of Orang Asli participants towards intestinal helminth infections with educational level and employment status

**Variable**	**Educational level**				**Employment status**			
	**Educated**	**Non educated**	**OR**	**95% CI**	**Working**	**not working**	**OR**	**95% CI**
**Attitude:**								
–Harmful to people’s health	43(33.3)	28(32.9)	1.0	0.6, 1.8	10(23.8)	61(35.5)	0.5	0.3, 1.2
**Practices:**								
–Washing hands before eating	66(50.8)	31(36.5)	1.9	1.2, 3.1*†	15(35.7)	82(47.4)	0.6	0.3, 1.2
–Washing hands after defecation	84(64.6)	50(58.8)	1.3	0.7, 2.2	27(64.3)	107(61.8)	1.1	0.6, 2.2
–Wearing shoes when outside the house	104(80)	65(76.5)	1.2	0.6, 2.4	35(83.3)	134(77.5)	1.4	0.6, 3.5
–Washing fruits before consumption	61(46.9)	27(31.8)	1.9	1.1, 3.4*†	23(54.8)	65(37.6)	2.0	1.0, 3.9*
–Washing vegetables before consumption	93(71.5)	55(64.7)	1.4	0.8, 2.5	30(71.4)	118(68.2)	1.2	0.6, 2.4
–Boiling drinking water	28(32.9)	31(23.8)	1.2	0.9, 1.6†	14(33.3)	45(26.0)	1.4	0.7, 2.9

All values are number (%). OR, Odds ratio. CI, Confidence interval. * Significant association (*P* < 0.05). † Variables were confirmed by multiple logistic regression analysis as significant associated factors.

### Associations of KAP of Orang Asli participants on intestinal helminth infections with helminth infection status among their children

Associations between the knowledge of respondents on intestinal helminths (signs and symptoms, ways of transmission and prevention) and the most common infections (trichuriasis, ascariasis and hookworm infections) among their children were not significant (*P*>0.05). However, Table 8 shows a significantly lower percentage of respondents who wear shoes/slippers when outside the house among children who were infected with *Trichuris* when compared to the non-infected children (72.8%; 95% CI= 62.6, 80.5 vs 87.0%; 95% CI= 81.4, 91.1). Similarly, significantly lower percentages of respondents who wash their hands before eating (32.4%; 95% CI= 24.3, 42.2 vs 51.4%; 95% CI= 44.7, 60.1) and after defecation (47.8%; 95% CI= 35.7, 57.1 vs 69.2%; 95% CI= 63.7, 78.7) was reported among *Ascaris*-infected children as compared to those who were not infected.

**Table 8:** Associations of attitude and practices of Orang Asli participants towards intestinal helminths with helminth infections status among their children **

**Variable**	**Trichuriasis**				**Ascariasis**				**Hookworm infection**			
	**Infected**	**Not infected**	**OR**	**95% CI**	**Infected**	**Not infected**	**OR**	**95% CI**	**Infected**	**Not infected**	**OR**	**95% CI**
**Attitude:**												
–Harmful to people’s health	51(33.1)	20(32.8)	1.0	0.5, 1.9	24(34.8)	47(32.2)	1.1	0.6, 2.1	10(31.3)	61(33.3)	0.9	0.4, 2.0
**Practices:**												
–Washing hands before eating	69(44.8)	28(45.9)	0.9	0.5, 1.7	22(32.4)	75(51.4)	0.5	0.2, 0.8*	10(31.3)	87(47.5)	0.5	0.2, 1.1
–Washing hands after defecation	93(60.8)	41(66.1)	0.8	0.4, 1.5	33(47.8)	101(69.2)	0.4	0.2, 0.7*	20(62.5)	113(62.1)	1.0	0.5, 2.2
–Wearing shoes when outside the house	115(72.8)	54(87.0)	0.4	0.2, 0.9*	57(82.6)	112(76.7)	1.4	0.7, 2.9	25(78.1)	146(86.4)	0.8	0.2, 1.4
–Washing fruits before consumption	63(40.7)	25(41.2)	1.0	0.5, 1.8	27(39.1)	61(41.8)	0.9	0.5, 1.6	14(42.4)	74(40.7)	1.1	0.5, 2.3
–Washing vegetables before consumption	104(68.0)	44(72.1)	0.8	0.4, 1.6	43(62.3)	105(71.9)	0.6	0.4, 1.2	24(75.0)	124(67.8)	1.4	0.6, 3.3
–Boiling drinking water	43(27.9)	16(26.2)	1.1	0.6, 2.1	24(34.8)	35(24.0)	1.7	0.9, 3.2	13(39.4)	46(25.3)	1.9	0.9, 4.2

All values are number (%). OR, Odds ratio. CI, Confidence interval. * Significant association (*P* < 0.05). ** Weighted data analysis.

### Multivariate analysis

The results of multiple logistic regression analyses for the factors significantly associated with the KAP on STH among the Orang Asli participants showed that the educational level of the respondents was the most important factor as it was significantly associated with many KAP items. The results indicated that respondents who had at least 6 years of formal education had significantly higher odds of hearing about intestinal worms (OR=2.1; 95% CI=1.3, 3.5), having knowledge on signs and symptoms (OR=1.7; 95% CI=1.1, 2.9), having knowledge on diarrhoea (OR=1.7; 95% CI=1.1, 2.9) and lack of appetite (OR=1.7; 95% CI=1.1, 2.9) as symptoms of STH infections, having knowledge on the prevention of STH infections (OR=1.7; 95% CI=1.1, 2.9), and having knowledge on taking de-worming drugs as a preventive measure (OR=1.7; 95% CI=1.1, 2.9) when compared with non-educated respondents. Regarding the practices towards STH infections, logistic regression output also confirmed the significant associations between the educational level and the hand washing before eating (OR=1.7; 95% CI=1.1, 2.9), washing fruits before consumption (OR=1.7; 95% CI=1.1, 2.9), and boiling drinking water (OR=1.7; 95% CI=1.1, 2.9).

Logistic regression analyses also showed that working respondents were at higher odds of having knowledge of transmission (OR=1.7; 95% CI=1.1, 2.9), and knowledge of stagnant water (OR=1.7; 95% CI=1.1, 2.9) and eating contaminated food (OR=1.7; 95% CI=1.1, 2.9) as the ways of STH transmission when compared with unemployed respondents. Similarly, the respondents from families with low household monthly income (<RM500) had significantly higher odds of having knowledge of abdominal pain (OR=1.7; 95% CI=1.1, 2.9) and eating contaminated food (OR=1.7; 95% CI=1.1, 2.9) when compared with respondents from families with higher household monthly income. It was also found that the male participants had significantly higher odds of washing hands before eating (OR=1.7; 95% CI=1.1, 2.9) and washing hands after defecation (OR=1.7; 95% CI=1.1, 2.9) than the females.

## Discussion

This is the first study in Malaysia on the knowledge, attitude and practices (KAP) of Orang Asli on soil-transmitted helminth (STH) infection*.* The results of the present study showed that knowledge about intestinal helminths among the participants was generally poor with low awareness about the symptoms, ways of transmission and preventive measures.

Our results showed that about 40% of the respondents had no prior knowledge of intestinal worms. This is in agreement with the findings from a recent study in western Cote d’Ivoire [24], where more than half of the respondents indicated being aware of the common intestinal helminth infections. However, a recent Zimbabwean study reported that only 26.2% of the grade 3 children in four selected schools expressed a prior knowledge on intestinal helminths [25]. However, a recent study among two communities in Rio de Janeiro, Brazil reported a higher awareness among their respondents [26]. Moreover, our findings showed that only one-tenth of the respondents mentioned some common intestinal worms by their names. These are contrary to the findings from a previous study in Nepal which demonstrated that the majority of their subjects had an excellent knowledge of intestinal helminths and this was attributed to the active health education programmes taking place in the study area [27]. This programme is however lacking in the communities where our study was conducted.

Moreover, the findings from this study showed that about a quarter of the respondents knew about the common signs and symptoms associated with intestinal helminth infections. This is in agreement with the Nepalese study which indicated that the knowledge of symptoms was described in general and vague terms by their subjects [27]. Conversely, similar studies from Cote d’Ivoire and Brazil demonstrated greater knowledge on the symptoms of intestinal helminths among their subjects [24,25]. This could be attributed to the nature of intestinal helminth infections which tend to be asymptomatic and remain in salience as chronic infections [28].

In our study, knowledge about transmission among the respondents showed little information as only about a quarter of them know about the ways of helminths transmission. This is in agreement with a previous study in rural Bangladesh [29]. It was surprising that in our study, none of the respondents mentioned open/indiscriminate defecation as a possible way of helminth infection transmission. Similarly, only 6% of the total respondents (about 19% of those had prior knowledge on worms) mentioned that playing with soil could be a possible way of acquiring helminth infection. In Cote d’Ivoire, soil was an unknown source of transmission for intestinal helminth infections while the majority of the respondents mentioned open defecation in water [24]. On the other hand, a previous study in Nepal showed that more than one-third of the respondents indicated playing with soil as a possible source of infection [27].

Our survey on the knowledge about prevention revealed that about 16% of the respondents had knowledge of some preventive measures such as washing hands before eating, wearing shoes when outside the house, boiling untreated drinking water, and use of de-worming drugs. Similar findings were reported in Zimbabwe [25] while higher level of knowledge on the preventive measures was reported in Egypt and western Cote d’Ivoire [18,24].

In the present study, the majority of the respondents who had prior knowledge on the intestinal worms thought that they are harmful to peoples’ health. A similar study in Brazil demonstrated that 95% of the respondents think that worms are harmful [26]. The fact that most of the respondents who know about worms also assumed that worms are harmful, showed that they most likely had this knowledge from health personnel in clinics/ hospitals or during community health campaigns. Interestingly, one of the respondents in our study had the opinion that worms are good for peoples’ health. On being questioned, she explained that a person with helminth infections will eat more than usual.

Regarding the practices towards helminth infections, we found that about two-thirds of the respondents practice hand washing before eating and after defecation. However, our findings regarding these practices among the children demonstrated a poor implementation on the daily practice as more than half and one third of the children living in these households do not wash their hands before eating and after defecation, respectively. The importance of hand washing in reducing the prevalence of STH infections is well documented [16,30]. However, a previous study in Zimbabwe showed that more than two-thirds of the respondents do not wash their hands before eating and after defecation [25]. On the other hand, a better situation was reported in Bangladesh where almost all of the participated children were said to wash their hands before eating and after defecation [29]. However, researchers should not always rely on the respondents’ answers to this kind of questions, and observations should be made.

In our study, about three-quarters and two-thirds of the respondents and their children commonly wear shoes/slippers when outside the house. This may explain the low prevalence of hookworm infections, which are transmitted by skin penetration by the infective larvae, among the children (17.6%). In rural Bangladesh, although most of the rural children had slippers many of them tend not to wear them when outside the house [29]. Moreover, we found that about half of the respondents and their children do not cut their fingernails regularly. This is in agreement with previous studies conducted in Bangladesh and Ethiopia [29,31]. While playing with soil, helminth eggs (*Ascaris* and *Trichuris*) tend to reside under the fingernails, and from there they are easily reintroduced into the body and start the infections. Thus, mothers and food handlers should keep their fingernails short and clean in order to prevent the parasites transmission to children and other people.

In the present study, more than two-thirds of the respondents do not boil their untreated drinking water. Similarly, only 14% of the participants in a previous study in Ethiopia boil their water before drinking [31]. The poor knowledge and practices towards avoiding the sources of STH infections among the participants in the present study could be attributed to general ignorance and lack of proper information through well coordinated health awareness programmes in Orang Asli communities. A promising finding about treatment-seeking behaviour was reported by the present study; almost all the participants seek treatment for abdominal pain and diarrhoea at the nearest clinic.

The findings of the present study showed that the males had more awareness of the intestinal helminths compared to the females and this could be attributed to the increased mobility of the males for work purposes, which provided them with more exposure to sources of knowledge. This low level of knowledge about intestinal helminths reported among the mothers may contribute to the high prevalence rate of STH among the studied children. In a focus group discussion in Cote d’Ivoire, none of the women who participated in a KAP survey was able to give an accurate description of signs of schistosomiasis [24].

Our findings showed that educated and working respondents had significantly better knowledge of the intestinal helminths, their signs and symptoms, ways of transmission and prevention, compared to the illiterate and unemployed respondents. Moreover, our findings confirmed that the educational level was the most important factor significantly associated with the KAP on STH among this population. In general, educated individuals are likely to be more exposed to information about intestinal helminths and good hygiene practices than illiterate individuals. Hence, our finding implies that improving the level of education in the community will probably increase the ability of people to better gain a wider understanding of intestinal helminth infections. A recent community-based study in Bangladesh showed that the educational level of the respondents positively associated with the knowledge and practices towards intestinal worms [32]. Similarly, a previous study showed that the better the educational level of the mothers, the lower the parasitic infection prevalence among their children in Iran [33]. On the other hand, a previous study found no significant association between the educational level of the respondents and their KAP on intestinal worms [24]. Nevertheless, a previous study on the KAP on malaria, measles, intestinal helminths, scabies, schistosomiasis and diarrhoea among two Kenyan communities showed that most of the respondents, particularly educated individuals, had knowledge on the intestinal helminths but compared with the other health problems, intestinal worms did not rank highly in people’s minds as an important health problem [28]. Compared to the asymptomatic and chronic nature of the intestinal helminth infections especially of low intensity, the acute and serious signs and symptoms caused by the other 5 diseases made people more aware of these diseases.

The present study showed that respondents from families with a household monthly income of ≥ RM500 were significantly more knowledgeable about the signs and symptoms, ways of transmission and prevention of intestinal helminths infections compared to those from poor families. Similarly, higher level of knowledge was also reported among the respondents from large size families (≥7 members) compared to those from smaller families. However, the majority of the respondents still do not take care to prevent transmission and this could be attributed to some economic limitations such as inability to buy shoes and soaps and a lack of proper sanitation facilities. Previous studies showed that children from lower-income families and with unemployed and less educated parents were at higher risk of STH than their counterparts [21,34].

With regard to STH situation among children living in these households, our findings suggest that by practising good hygiene measures (such as hand washing and wearing shoes when outside the house) heads of families are more likely to protect their children and other family members from acquiring STH infections. Hand washing, especially by mothers and food handlers will help in preventing food contamination and will eventually prevent STH transmission. On the other hand, those who walk barefooted may acquire the infections and indirectly become a source of infection to other family members. Since the significant association of wearing shoes was reported with trichuriasis and ascariasis, the mechanism here could be mechanical transmission by bringing the STH infective stages (ova) on the dirty feet into the house. In Egypt, a significant reduction in risk of STH infections has been reported among children of mothers who practise hand washing after defecation [18].

The present survey provides a community-based picture of the KAP on STH among Orang Asli people in Lipis, Pahang. Poverty and underdevelopment are the predominant features of Orang Asli communities, and education levels still lag far behind those achieved by other communities. Moreover, several studies have indicated a continued high prevalence of STH among these people. Hence, we believed that our results are generalisable to the entire Orang Asli population in Malaysia. However, further studies among other rural Malaysian populations are required.

## Conclusions

This study reveals inadequate knowledge, attitude and practices towards intestinal helminth infections among Orang Asli, which probably leads to the continued high prevalence and intensity rates of infections. These findings, coupled with the high prevalence and associated key factors reported in the first part of this study provide firm evidence that there is a great need for proper health education programme and community mobilization to enhance prevention and instil better knowledge on STH transmission and prevention in these communities. Successful efforts to eliminate STH will improve the nutritional and health status of children as well as contribute to higher educational attainment, productivity, income and quality of life among Orang Asli communities.

## Competing interests

The authors have declared that no competing interests exist.

## Authors’ contributions

NAN was involved in all phases of the study, including data collection, data analysis, interpretation, and write-up of the manuscript; AB and HMA designed and supervised the study, and revised the analysis and manuscript. AA and MAR were involved in the collection and laboratory examination of samples. All authors read and approved the final manuscript.
